# Ultrasound in Microsurgery: Current Applications and New Frontiers

**DOI:** 10.3390/jcm13123412

**Published:** 2024-06-11

**Authors:** Rachel Cowan, Gursimran Mann, Ara A. Salibian

**Affiliations:** Division of Plastic and Reconstructive Surgery, University of California, Davis School of Medicine, Sacramento, CA 95817, USA

**Keywords:** microsurgery, plastic and reconstructive surgery, ultrasound, free flap surgery, perforator flaps, flap design, lymphovenous bypass, lymphovenous anastomosis, lymphedema

## Abstract

Ultrasound has revolutionized reconstructive microsurgery, offering real-time imaging and enhanced precision allowing for preoperative flap planning, recipient vessel identification and selection, postoperative flap monitoring, and lymphatic surgery. This narrative review of the literature provides an updated evidence-based overlook on the current applications and emerging frontiers of ultrasound in microsurgery, focusing on free tissue transfer and lymphatic surgery. Color duplex ultrasound (CDU) plays a pivotal role in preoperative flap planning and design, providing real-time imaging that enables detailed perforator mapping, perforator suitability assessment, blood flow velocity measurement, and, ultimately, flap design optimization. Ultrasound also aids in recipient vessel selection by providing assessment of caliber, patency, location, and flow velocity of recipient vessels. Postoperatively, ultrasound enables real-time monitoring of flap perfusion, providing early detection of potential flap compromise and improved flap survival rates. In lymphatic surgery, ultra-high frequency ultrasound (UHFUS) offers precise mapping and evaluation of lymphatic vessels, improving efficacy and efficiency by targeting larger dilated vessels. Integrating ultrasound into reconstructive microsurgery represents a significant advancement in the utilization of imaging in the field. With growing accessibility of devices, improved training, and technological advancements, using ultrasound as a key imaging tool offers substantial potential for the evolution of reconstructive microsurgery.

## 1. Introduction

The evolution of reconstructive microsurgery has tremendously benefited from parallel growth and incorporation of imaging technologies into the field. Over the last several decades, multiple imaging modalities have allowed for improvement in microsurgical procedures, particularly within the realm of preoperative planning. High-resolution computed tomography (CT) scans led to the introduction of virtual surgical planning (VSP) and custom patient-specific hardware in head and neck reconstruction [1,2,3,4]. CT and magnetic resonance angiography have played critical roles in flap planning, particularly for autologous breast reconstruction procedures [5,6,7,8,9]. Formal angiography has also been instrumental in microsurgical lower extremity limb salvage, particularly in patients with vascular comorbidities [10,11].

Ultrasound, often associated as only a diagnostic modality and once foreign to the reconstructive microsurgeon, has more recently emerged as a versatile and essential tool in microsurgery. Duplex ultrasound uses high-frequency sound waves to image structures and assess blood flow, performed in the B-mode and Doppler mode of ultrasonography, respectively. In B-mode, real-time grey-scale images can be obtained of multiple important anatomic structures in microsurgery including superficial and deep fascial layers, muscle, fat layers, arteries, veins, and perforating vessels. As these structures of interest in reconstructive microsurgery are relatively superficial to other anatomy traditionally visualized by ultrasonography (solid and hollow viscera), higher-frequency ultrasound probes (with inversely correlated tissue depth) have become the mainstay. Conventional high-frequency probes typically range between 15 and 20 MHz and clinically approved ultra-high frequency ultrasound (UHFUS) probes have upper ranges of 70 MHz [12,13]. Furthermore, color doppler ultrasound (CDU) allows for the color overlay of vessel flow to be able to three-dimensionally localize both major and perforating vessels up to the subdermal capillary layer while simultaneously assessing flow. The integration of ultrasound into microsurgical practice offers surgeons non-invasive, high-resolution imaging capabilities and real-time visualization and guidance, augmenting the ability to assess critical vascular anatomy for several facets of microsurgery. 

Ultrasound technology has likely had the most meaningful impact on free tissue transfer, the cornerstone of microsurgery, that involves transplanting tissue from one area of the body to another with anastomosis of small donor and recipient vessels to allow for the needed tissue viability, form, and function at the recipient site. Whether for wound coverage, breast reconstruction, or limb salvage, successful flap surgery hinges on meticulous planning, precise execution, and vigilant postoperative monitoring. Ultrasound has proven to be an indispensable tool at multiple stages of free flap procedures including preoperative flap planning and design, recipient vessel selection, and postoperative flap monitoring [8]. In this setting, CDU provides a cost-effective means of real-time and detailed data acquisition on vessel anatomy, size, trajectory, and flow velocity without radiation exposure. Additional areas of microsurgery and supermicrosurgery that have benefited from ultrasound technology include lymphatic surgery. Ultra-high frequency ultrasound has become a highly useful tool in preoperative planning for lymphovenous bypass (LVB) given its ability to accurately detect lymphatic vessel size and quality in addition to surrounding venules [14]. The purpose of this review is to summarize the established and emerging applications of ultrasonography in reconstructive microsurgery to better define the scope of this tool in current practice as well as the potential future directions for its utilization.

## 2. Materials and Methods

A comprehensive review of the literature was performed utilizing PubMed databases from 2000 to 2023, with keywords and MeSH terms “microsurgery”, “plastic and reconstructive surgery”, “ultrasound”, “free flap surgery”, “perforator flaps”, “flap design”, “lymphovenous bypass”, “lymphovenous anastomosis”, and “lymphedema”. The retrieved articles were then screened based on predefined inclusion and exclusion criteria to select those that meet the objectives of the review. Inclusion criteria included observational and experimental original articles and systematic reviews published in peer-reviewed journals, directly addressing the use of ultrasound in microsurgery within the field of plastic and reconstructive surgery. The studies reporting on applications, techniques, outcomes, and advancements related to ultrasound imaging were reviewed. Exclusion criteria included non-English manuscripts and abstracts, studies not specific to microsurgery, non-human subjects, non-original and non-peer-reviewed sources, as well as studies with inadequate methodology and outdated information. References from identified articles were additionally queried for relevant studies.

## 3. Discussion

Ultrasound has well-described applications in reconstructive microsurgery. The relevant literature highlights ultrasonography as a valuable tool for multiple aspects of microsurgery including the entire spectrum of preoperative, intraoperative, and postoperative care. Most notably, ultrasound has proven beneficial in preoperative planning, as with other imaging modalities, for identifying and assessing donor vessels for perforator flaps as well as evaluating recipient vessels for free tissue transfer. Additional intraoperative and postoperative applications for ultrasound in free tissue transfer have also been described. Utilization of high-frequency ultrasound and CDU in free tissue transfer is highlighted given the ability to provide real-time, color-encoded, and in-depth visualization of blood vessels overlying gray-scale B-mode tissue images [15,16,17]. Ultra-high frequency ultrasound has increasingly become a useful tool in lymphatic surgery, particularly LVB, due to the need for high resolution and shallow depth visualization. These different applications and the corresponding evidence were reviewed within the relevant clinical scenarios. 

### 3.1. Perforator Flap Planning and Design

**Figure 1:**
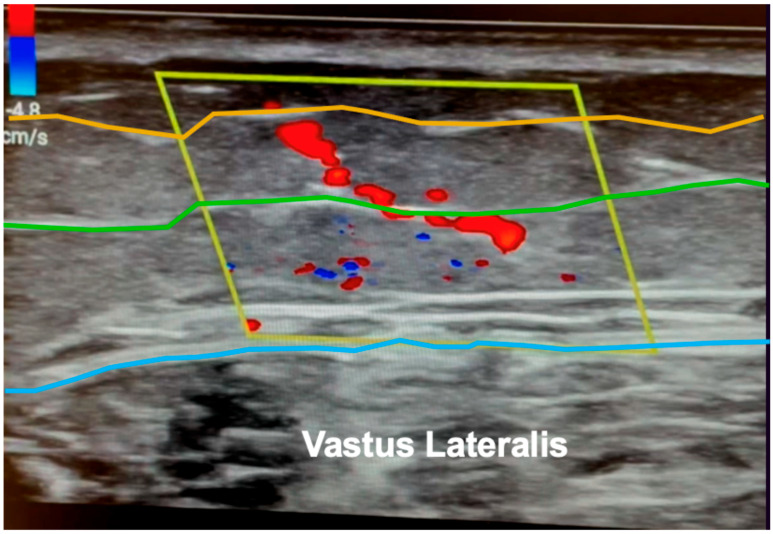
Color duplex ultrasonography (CDU) of an anterolateral thigh perforator. The deep fascia (*blue line*) corresponding to the traditional plane of dissection, superficial fascia (*green line*) corresponding to the thin plane of dissection, and subcutaneous plane (*orange line*) corresponding to the superthin plane of dissection are highlighted. The course of a perforator traversing the subcutaneous tissue and superficial fascial planes is easily visualized in *red*. Imaging performed by senior author A.A.S.

**Figure 2:**
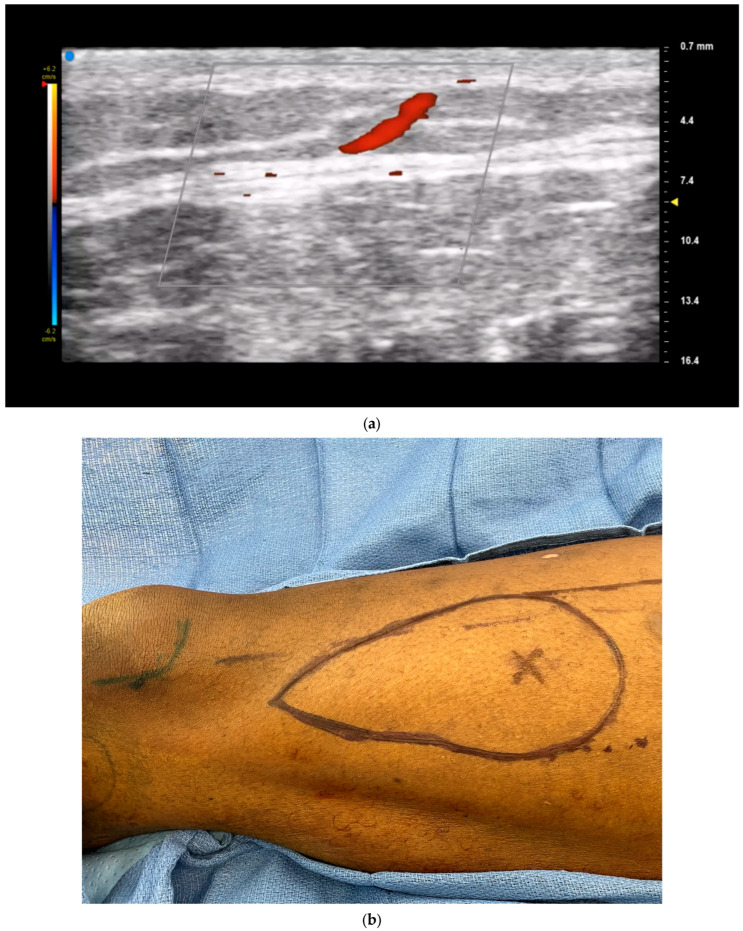

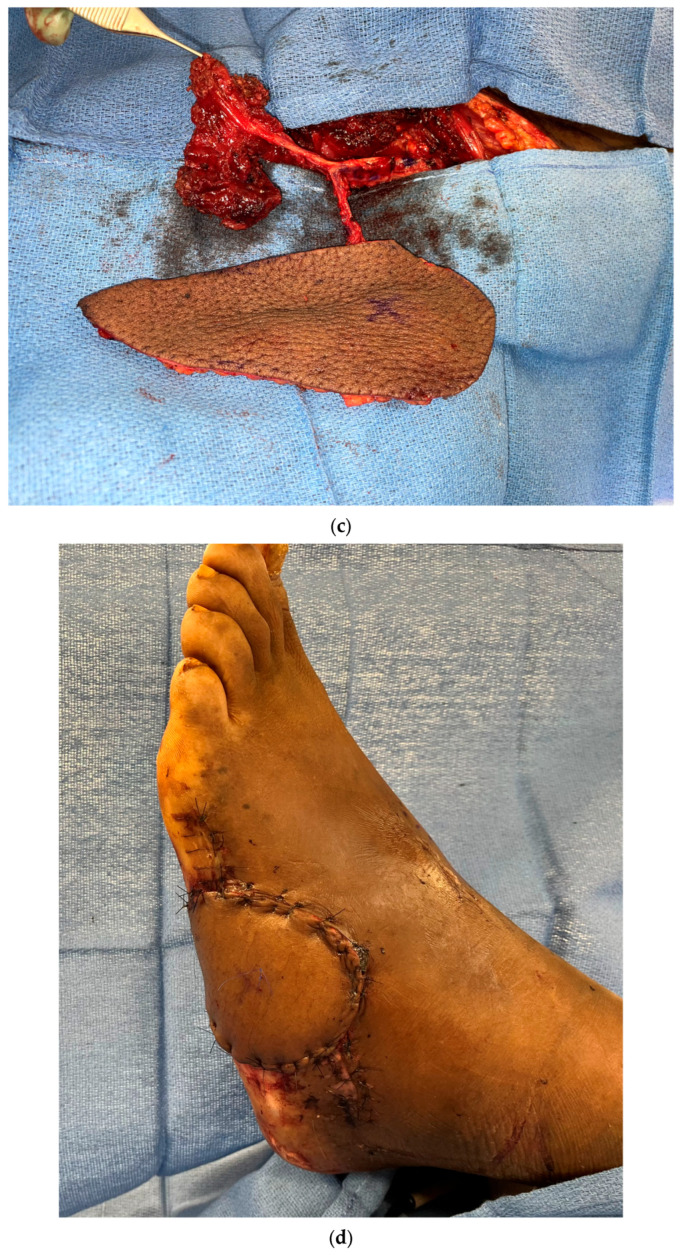

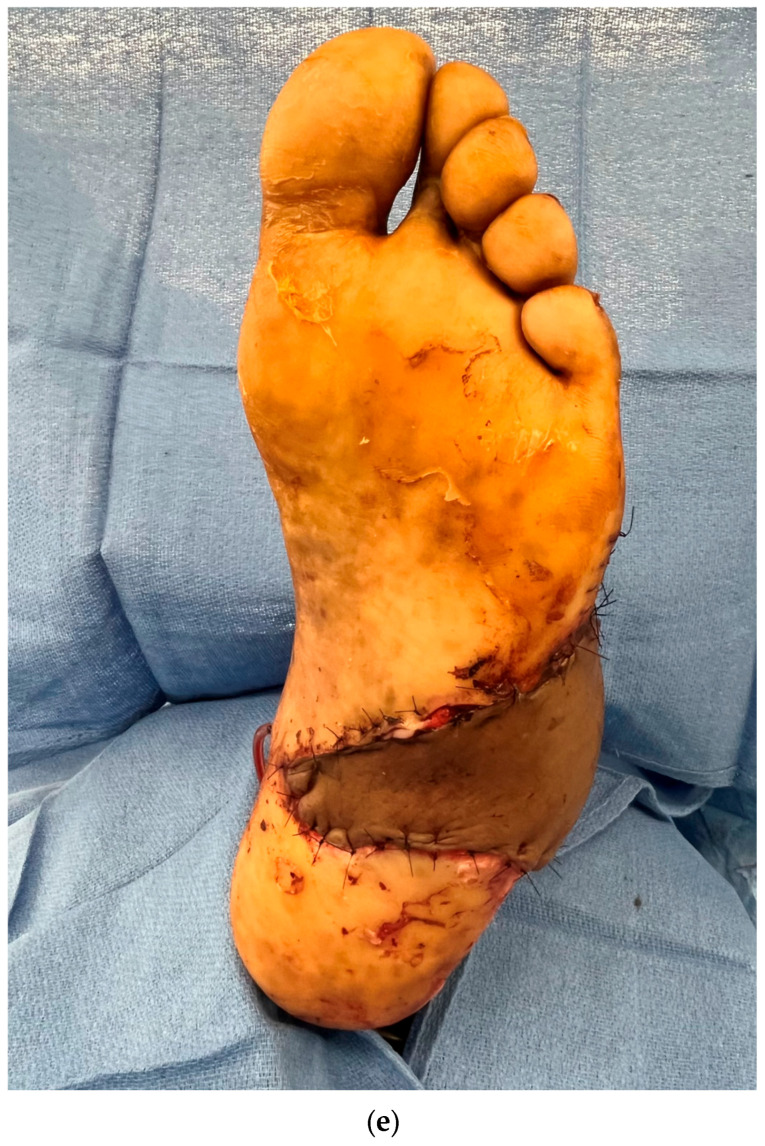
Chimeric suprafascial anterolateral thigh (ALT) flap for lateral foot reconstruction. CDU identification of perforators (**a**) allows for precise flap design (**b**) and elevation (**c**). Final flap inset (**d**,**e**). Patient of senior author A.A.S.

Color doppler ultrasonography is particularly helpful for free tissue transfer planning. A recent systematic review of ultrasound in microsurgical reconstruction demonstrated that nearly 40% of the included studies utilized ultrasound for mapping of perforators [8]. In this regard, CDU provides detailed, real-time information on perforator anatomy and flow and can also reveal unexpected anatomical variations or aberrations that might impact flap selection [17,18]. As high-frequency ultrasound can detect sub-millimeter vessels, it is well suited for imaging smaller perforating vessels in addition to named axial vessels [7]. The exact path of the perforator beneath the fascia (whether septal, intramuscular, or a mixed course) can be evaluated up to the originating vessel, thereby accurately outlining the dissection path to be followed during the operation (Figure 1). Unlike acoustic Doppler, CDU also provides visualization of the exact location of the perforator in relation to, and differentiated from, the source vessel. Identification of these perforator locations then aids in flap design by allowing for templated dissections around known locations rather than large exploratory incision (Figure 2), aiding in flap design optimization [16]. 

**Figure 3:**
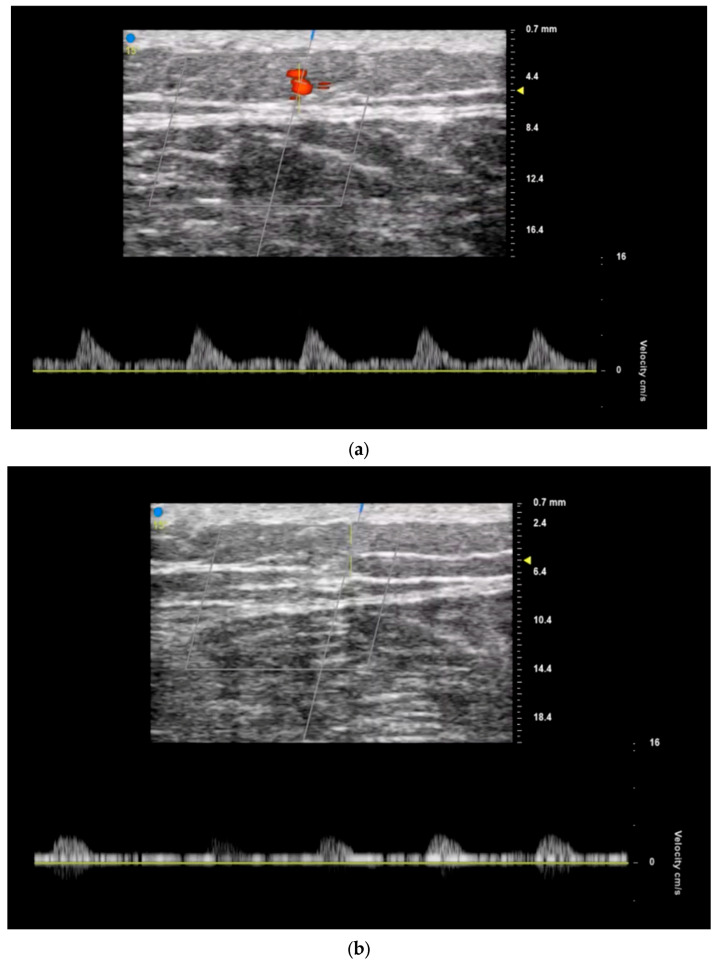
Perforator flow velocity assessment allows for selection of optimal vessels when designing flaps. Anterolateral thigh perforator with (**a**) higher flow velocity and (**b**) lower flow velocity. Patient of senior author A.A.S.

Surgeons can also evaluate perforator suitability for flap transfer by using ultrasound to quantify the blood flow velocity (or peak systolic flow velocity) of individual perforators (Figure 3). Multiple studies have shown a positive correlation between peak systolic flow velocity and successful perforator dissection and reconstruction [17,18,19]. A study by Yoshida et al. demonstrated a strong correlation between the success of perforator dissection and the preoperative peak systolic flow velocity in the perforator [17]. The likelihood of successful dissection was greatest when the peak systolic flow velocity in the perforator was at least 20.0 cm/s. Compared to perforators with velocities of 15.0–19.9 cm/s or less than or equal to 14.9 cm/s, those with a velocity of 20.0 cm/s or more had a higher success rate. The Flap Viability Index (FVI) can also be measured, which is an empirically based equation that considers perforator diameter and flap weight. A study by Dusseldorp et al. demonstrated that if the FVI exceeds 10, total flap survival is likely; if under 10, partial flap necrosis is probable [19]. This evaluation helps guide the choice of donor site and selection of optimal perforators. Ultrasound can also help evaluate the thickness of the flap, which is crucial for functional and cosmetic outcomes. For thin flaps, identifying the superficial fascial plane using ultrasound as well as the branching pattern and course of the perforator through these fascial layers can also aid in dissection by serving as a visual guide [20]. 

**Figure 4:**
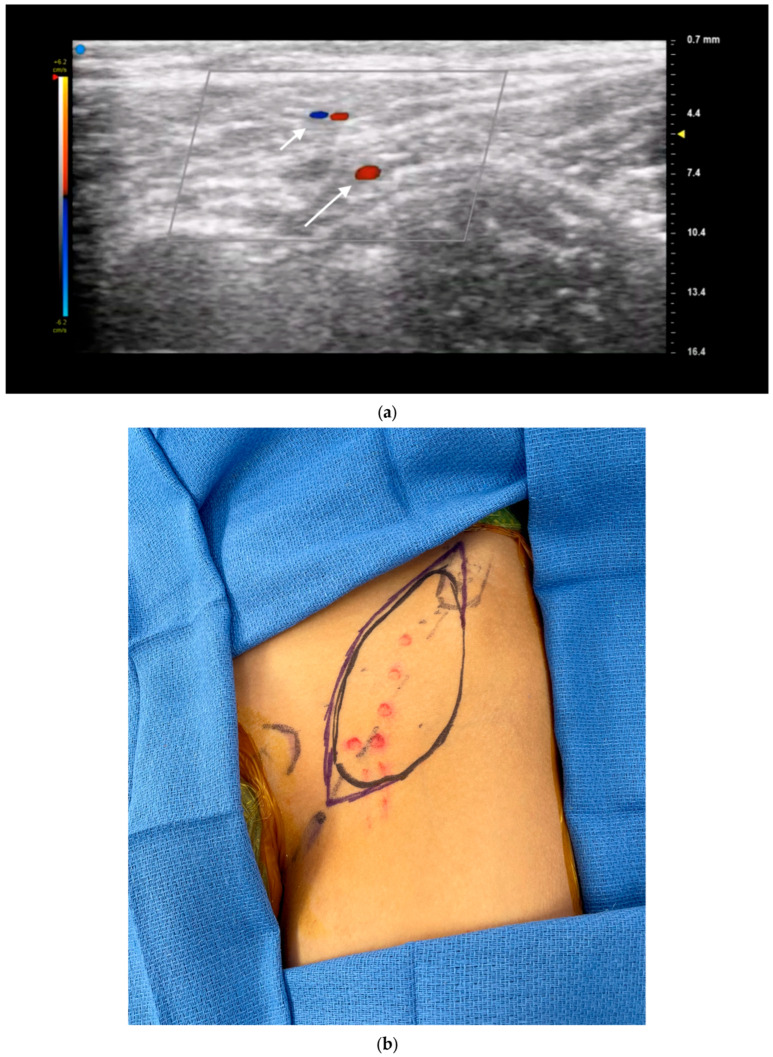

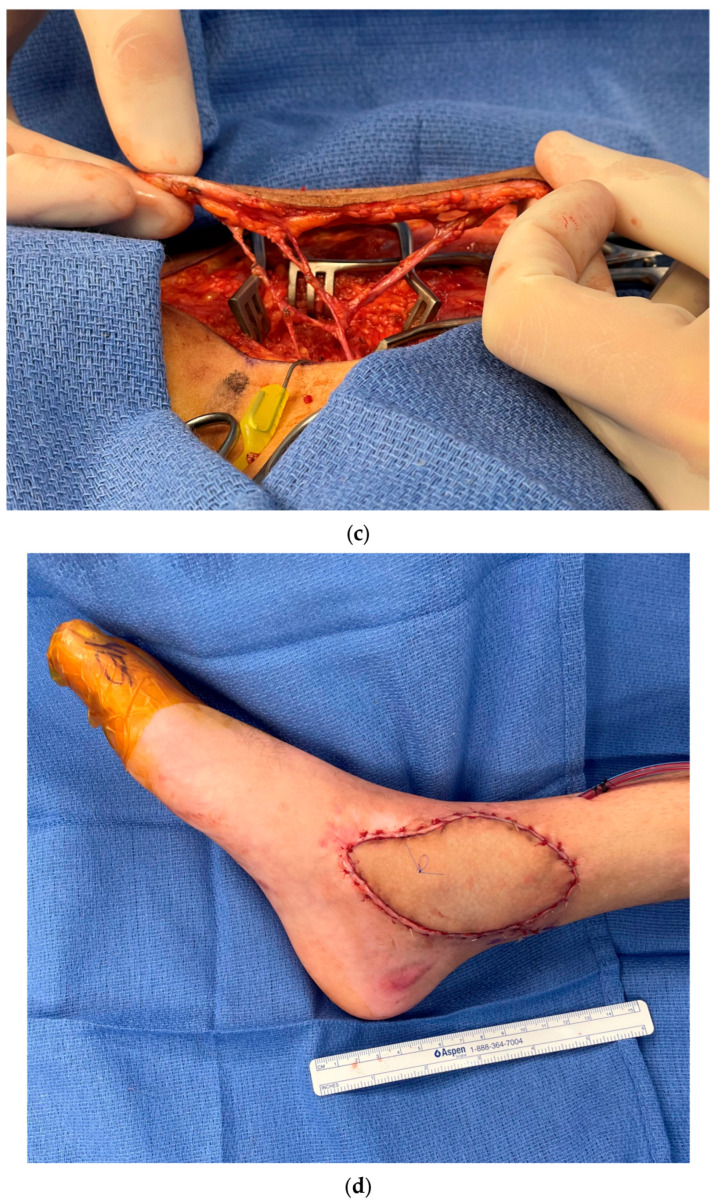

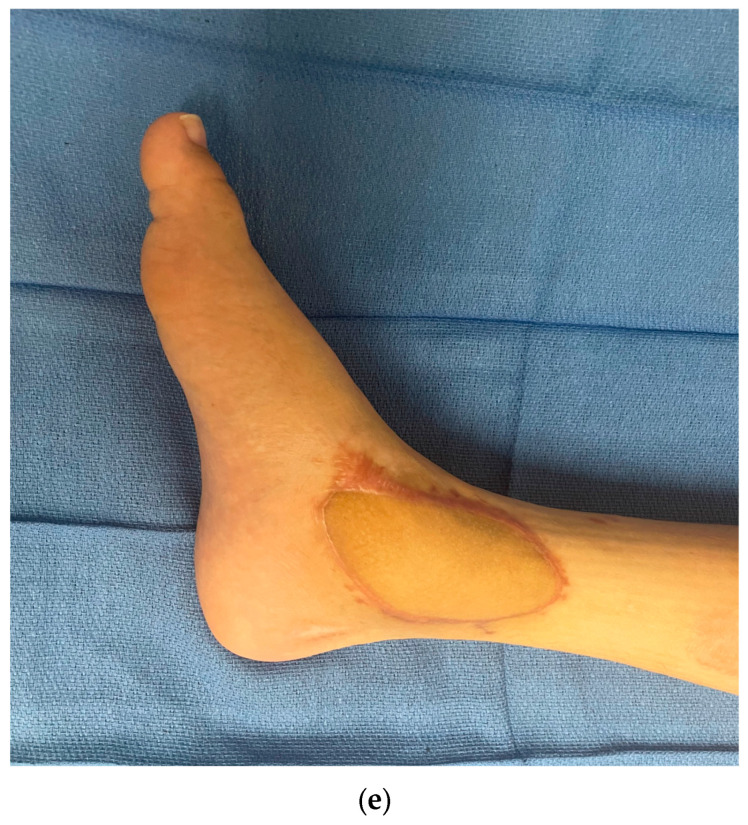
Color duplex ultrasonography facilitates planning of thin flaps through precise perforator location. (**a**) CDU mapping of thin SCIP flap demonstrating superficial branch (*short arrow*) and deep branch (*long arrow*). (**b**) Flap design based on CDU mapping. (**c**) Completion of flap elevation above level of superficial fascia. Postoperative photos (**d**) immediately and (**e**) 6 months after surgery. Patient of senior author A.A.S.

Color doppler ultrasound has been described in preoperative planning for multiple different flaps including deep inferior epigastric perforator (DIEP), superficial inferior epigastric artery (SIEA), superficial circumflex iliac artery perforator (SCIP), anterolateral high (ALT), thoracodorsal artery perforator (TDAP), and profunda artery perforator (PAP) flaps [8]. Certain flaps also have unique features that ultrasound is also able to address independently. For example, the anatomy of SCIP flaps can be complex and highly variable, making preoperative flap planning and design more challenging. Ultrasound can help identify and evaluate the superficial circumflex iliac artery (SCIA) branches and individual medial and lateral perforators in real time to allow for appropriate design of thin and superthin flaps (Figure 4). Prior research has shown a 100% correlation between the number and emergence points of the perforators detected by preoperative mapping via ultrasound and intraoperative anatomy [21].

Two studies by Visconti et al. demonstrated that UHFUS is especially useful when planning for thin, superthin, and pure skin perforator flaps, as it can help further characterize the anatomy of the subcutaneous tissue and its microvascular network [20,22]. UHFUS enables the visualization of microanatomical structures as small as 30 µm, providing a more comprehensive view of microvascular structures and subcutaneous anatomy compared to HFUS. The 48 MHz probes allow for much clearer visualization of superficial, small perforator vessels through the superficial fascia and the subcutaneous fat, allowing for safer dissection at this level. Visconti et al. demonstrated a 100% correlation between the preoperative UHFUS results and intraoperative findings during SCIP dissection [20].

At many institutions, CDU is the predominant modality used for preoperative perforator imaging in flap surgery, in addition to CT angiography (CTA) and MR angiography (MRA) [8,23,24]. When comparing CDU to other imaging modalities such as CTA and MRA, ultrasound has certain advantages in preoperative planning including real-time image acquisition, assessment of blood flow velocity, and ease of examination [7,8,25]. CDU also avoids ionizing radiation and contrast agents, is less time consuming, and requires less specialized equipment and expertise. Kehrer et al. compared CDU, handheld Doppler, and CTA and demonstrated that CDU had the highest sensitivity (95.7%) and the highest positive predictive value (94.3%) in identifying ALT perforators [16]. Another study by Mijuskovic et al. examining imaging modalities in 125 DIEP flaps found a significantly stronger correlation between color doppler ultrasound and intraoperative findings of perforator detection, size, and selection when compared with CTA data. Additionally, if none of the preoperative imaging studies matched intraoperative perforator selection, there was an association with a higher incidence of flap loss [25]. The ability of ultrasound to readily assess the size of perforator vein has also been cited as a potential benefit for DIEP flap planning [26].

Intraoperative applications for ultrasound have also been described for flap planning. Song et al. demonstrated that the direction of rotation of propeller flaps can significantly alter flow velocity and volume [27]. Using this intraoperative assessment, the authors choose clockwise or counterclockwise rotation arcs for propeller flaps based on the direction with the maximum flow [8].

### 3.2. Recipient Vessel Selection

Recipient vessel availability and quality are critical in planning microvascular reconstruction around the body [28,29,30]. Particularly, in the setting of traumatic injuries or wounds complicated by radiation or systemic factors such as diabetes, options for recipient vessels can be limited, posing a significant challenge for free tissue transfer [29,30]. Color doppler ultrasound has emerged as an important tool to help surgeons with recipient vessel selection for free tissue transfer. High-frequency probes are commonly utilized for recipient vessel identification to balance image resolution with depth. While UHFUS provides much greater resolution, depth becomes limited with higher frequencies, which reduces its efficacy when imaging axial vessels deeper in the tissue compared to perforators. 

Multiple imaging modalities can be utilized for recipient vessel evaluation in addition to clinical examination, including CT angiography, formal angiography, and acoustic Doppler. Color duplex ultrasonography is particularly useful for recipient vessel evaluation, as it provides the exact location of the desired structure including the depth in real time and provides information on vessel size and velocity. This becomes particularly useful in vessel depleted recipient areas that require use of unnamed vessels or perforators for anastomosis in a freestyle fashion. 

Identifying appropriate recipient veins can be particularly challenging in compromised recipient sites, especially if vena comitans are too small or additional venous drainage is needed. CDU can help identify additional superficial veins within or near the defect as potential sources of outflow. Alternatively, the flap itself might have a superficial vein that has dominant drainage. In such situations, identifying and utilizing a superficial vein becomes valuable for ensuring adequate flap drainage [31].

Another important way in which CDU aids in recipient vessel selection is by assessing the blood flow velocity and flow volume of the recipient vessel. The velocity of the recipient serves as an important and dependable parameter that can influence the adequacy, and thus choice, of the recipient vessel. This becomes particularly important in perforator-to-perforator anastomoses and smaller vessels with reduced flow. A study by Hong et al. suggested that the minimum perforator flow velocity to ensure adequate blood flow to the skin flap is approximately 15 to 20 cm/s [30,32,33]. Color duplex ultrasonography therefore provides valuable information for recipient vessel evaluation, beyond just patency and size, that can further aid in decision-making for recipient vessel selection.

### 3.3. Postoperative Monitoring 

Postoperative monitoring of free flaps for potential arterial or venous compromise is a critical component of free tissue transfer procedures. Unless these complications can be successfully salvaged, achieving a high success rate remains challenging [34,35]. A study by Shinomiya et al. found that revascularization surgeries performed within 4 h of diagnosing vascular compromise achieve a higher salvage rate compared to those conducted at 9 h, underscoring the importance of early, accurate detection of postoperative flap complications [34]. Currently, postoperative tissue perfusion is typically monitored clinically by assessing flap color, temperature, edema, turgor, capillary refill, and bleeding. However, while clinical monitoring does have a relatively high success rate, relying solely on this method for monitoring is imprecise and may increase the risk of misdiagnosis and flap failure [36,37]. 

Multiple adjunctive means of postoperative flap monitoring are routinely employed including acoustic Doppler, buried flap monitoring devices, and other non-invasive devices [38,39,40]. Ultrasound serves a critical function in the postoperative period of flap surgery to monitor for adequate flap perfusion and the development of hematomas, seromas, or other skin or soft tissue infections [36]. High-frequency ultrasound is another tool that can assist with assessment of postoperative tissue perfusion and flap viability and detect signs of vascular compromise in real time. Color duplex ultrasonography allows for the evaluation of both arterial and venous flow within a flap postoperatively, in addition to flow velocity and assessment of thrombosis that uniquely provides a multifaceted assessment of postoperative flap perfusion [37,41]. Vigilant monitoring of the vascular anastomotic pedicle using ultrasound facilitates the prompt return of patients experiencing vascular compromise to the operating room. This timely intervention allows for potential flap salvage. In a study by Shinomiya et al., and another by Gonzalez et al. that examined the use of ultrasound postoperatively to detect vascular compromise in flaps that necessitated re-exploration, ultrasound findings precisely aligned with the intraoperative observations [34,37]. Ultrasound was able to differentiate between arterial and venous thromboses, with a false-positive rate of 0%, with complete salvage achieved in all cases [34]. Ultrasound also serves as a valuable tool for guiding postoperative interventions, such as drainage procedures or secondary revisions [36]. Overall, the utilization of ultrasound in the postoperative phase of microsurgery enhances patient care by providing clinicians with timely and accurate information to guide clinical decision-making and optimize salvage when needed.

### 3.4. Lymphatic Surgery

Lymphovenous bypass surgery (LVB), also known as lymphovenous anastomosis (LVA), has become an established supermicrosurgical technique for the treatment of lymphedema through delayed lymphatic reconstruction as well as prevention of lymphedema through immediate lymphatic reconstruction [42,43,44,45,46,47,48]. Multiple imaging techniques are utilized throughout the diagnosis and workup of lymphedema patients including lymphoscintigraphy, indocyanine green (ICG) lymphography, magnetic resonance lymphography, and ultrasound [49,50,51,52,53,54]. Indocyanine green lymphography, first described for lymphedema evaluation in 2007, has become the most utilized imaging modality both for lymphedema evaluation, operative decision-making, and preoperative planning of LVB [54,55,56,57,58,59]. While ICG lymphography remains the gold standard, it has certain limitations including the inability to detect lymphatic channels deeper than 1.5 cm or those concealed by dermal backflow as well as contraindication in patients with iodine allergy [14,53,60,61,62]. Prior studies have also shown that ICG lymphography does not provide clinically significant images in areas of sclerotic skin or lymphatic vessels or in patients with late-stage lymphedema [14,52,62].

**Figure 5:**
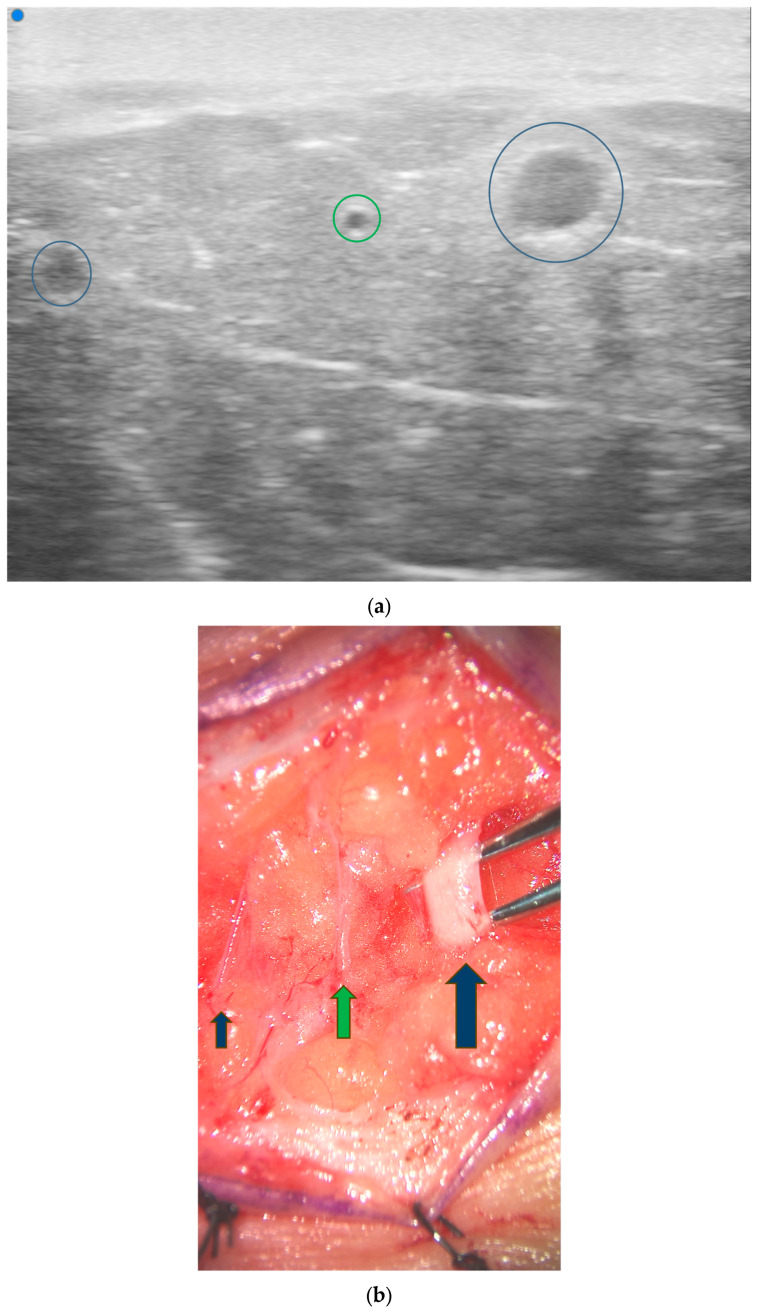
(**a**) Ultra-high frequency ultrasound demonstrating lymphatic (*green circle*) with small venule on the left (*small blue circle*) and large venule on its right (*large blue circle*). (**b**) Intraoperative photograph during lymphovenous bypass surgery demonstrating exact correlation of ultrasound to anatomic structures including lymphatic channel (*green arrow*), with small and large veins on the left and right, respectively (*small blue arrow, large blue arrow, respectively*).

Ultra-high frequency ultrasound is a valuable tool in the planning and execution of LVB, as it provides detailed insight into lymphatic architecture both as an adjuvant to ICG lymphography and, in certain cases, as an alternative. The advent of UHFUS, operating at frequencies as high as 70 MHz and with a resolution of 30 μm, has significantly improved the detection and visualization of pertinent anatomical structures. Hayashi et al. demonstrated this improvement over traditional high-frequency ultrasound in 2019, including an increased number of lymphatic vessels identified with an overall greater diameter using UHFUS [12]. Similar to perforator mapping, UHFUS enables precise localization of lymphatic vessels and nearby venules to optimize both efficiency and efficacy in lymphovenous bypass (Figure 5). 

**Figure 6:**
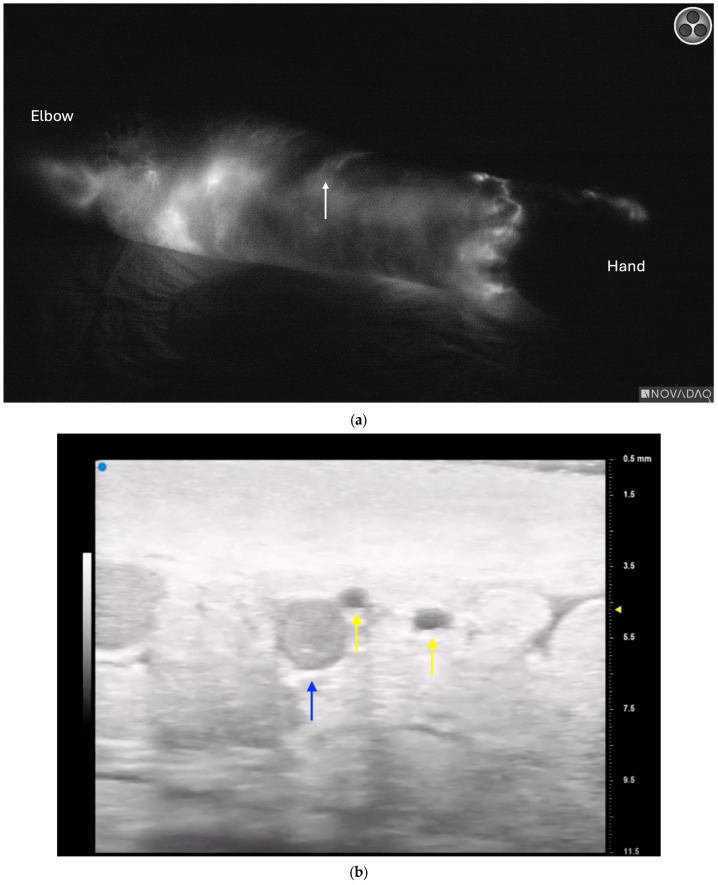
Ultra-high frequency ultrasound can identify lymphatic channels in more advanced lymphedema patients. (**a**) ICG lymphography of upper extremity demonstrating diffuse dermal backflow with splash pattern distally. Dilated lymphatic channels identified within backflow (*white arrow*) using UHFUS (**b**), which demonstrated two lymphatic channels (*yellow arrows*) and nearby venule (*blue arrow*). Patient of senior author A.A.S.

UHFUS has also proven effective in addressing the limitations of ICG-lymphography, as it can detect lymphatic vessels that are concealed by dermal backflow patterns or situated in deeper tissue layers (Figure 6) [12]. This has expanded the indications for LVB, which is traditionally only considered for earlier stage disease with linear patterns on ICG-lymphography [61]. Visconti et al. demonstrated successful outcomes with LVB in 45 advanced cases where lymphatic channels were unable to be detected either by ICG-lymphography or lymphoscintigraphy [62].

**Figure 7:**
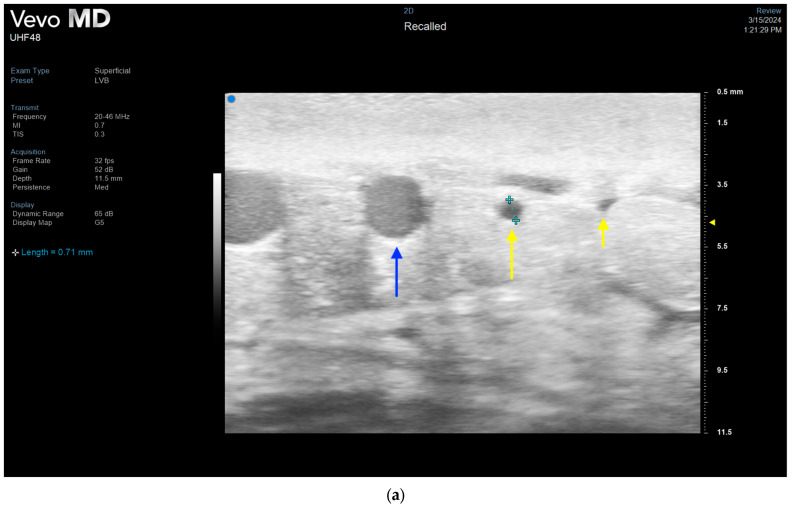

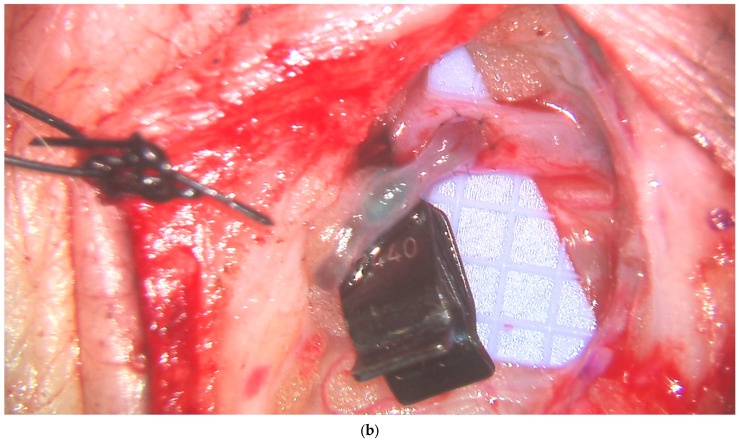
Ultra-high frequency ultrasound optimizes preoperative LVB planning by allowing for selection of more dilated lymphatics not discernible on ICG lymphography. (**a**) Dilated, 0.71 mm lymphatic is seen on UHFUS (*long yellow arrow*) next to a smaller, sclerotic lymphatic (*short yellow arrow*) and venule (*blue arrow*). (**b**) End-to-side lymphovenous anastomosis of larger lymphatic demonstrated in ultrasound. Patient of senior author A.A.S.

UHFUS also allows for further optimization of LVB by visualization of actual lymphatic vessel size and quality [12]. While linear channels on ICG are helpful in detecting the position of underlying lymphatic channels, they typically do not reveal any information about the degree of lymphosclerosis or the lymphatic channel size, unless terminally diseased to the point of occlusion. UHFUS, on the other hand, can accurately assess the size of lymphatic channels in real time, allowing for selection of larger lymphatic vessels (Figure 7) [12,14]. It additionally can be utilized to assess the thickness of the lymphatic vessel wall in relation to the lumen to help the surgeon determine the degree of lymphosclerosis. Bianchi et al. demonstrated good correlation of UHFUS images of lymphatic vessels and histological analysis confirming the ability of UHFUS to accurately assess lymphosclerosis [14]. As functioning lymphatics are one of the most critical aspects in the efficacy of LVB, the ability of UHFUS to provide this information for preoperative planning is invaluable. Finally, ultrasound enables visualization of venous branching patterns and even valves to allow for the selection of optimal recipient venules to minimize venous backflow [53,62,63].

### 3.5. Future Directions and Additional Considerations

As the utilization of ultrasound in reconstructive microsurgery increases and ultrasound technology improves, its application will continue to evolve within the field. Several exciting frontiers are emerging that show promise in enhancing the current uses of ultrasound in terms of addressing limitations while also expanding applicability. 

Contrast-enhanced ultrasound (CEUS) combined with 3D image reconstruction has the ability to improve spatial context in ultrasonography [7]. CEUS involves injecting microbubble contrast agents intravenously, enhancing the contrast and quality of ultrasound images [64]. When combined with 3D reconstructed images, CEUS provides enhanced visualization of tissue depth and contour in relation to the relevant vascular anatomy of donor and recipient sites and can provide more accurate and precise information on the quality, number, course, and location of perforators [7]. Particularly in the field of supermicrosurgery, thin flaps, and freestyle flaps, CEUS holds significant promise in improving the accuracy of preoperative planning for these procedures. 

B-flow contrast enhanced ultrasound (BCEUS) is another innovative technique for preoperative perforator mapping by further improving the accuracy of small vessel visualization. A study by Heneweer et al. comparing BCEUS with contrast enhanced ultrasound (CES), color Doppler ultrasound (CDUS), and B-flow ultrasound (BUS) demonstrated that only BCEUS could accurately map the epifascial and subcutaneous course with a clear delineation and a precision of 4 mm [65]. Zinser et al. also demonstrated that BCEUS, when combined with CDUS, can be a viable supplement or even an alternative to CTA for the visualization of perforator fascial penetration, subcutaneous plexus, and skin point [66]. The use of BCEUS for surface navigation enabled accurate perforator mapping with a sensitivity of 91.2% and a specificity of 88.9%. Moreover, BCEUS was found to be more precise and beneficial than CTA for fascial penetration due to the real-time control provided during ultrasound as well as the ability to assess dynamic vessel parameters, such as the trajectory of blood flow. 

Artificial intelligence (AI) is another powerful tool that can be combined with ultrasound technology. Algorithms can analyze vast amounts of ultrasound data, identifying subtle patterns and predicting outcomes. In breast reconstruction, researchers are currently exploring the use of AI systems to analyze ultrasound images and identify potential anomalies such as tumors or vascular abnormalities, as well as to assist with preoperative planning, intra-operative decision making and precision enhancement, and postoperative monitoring [67]. Advanced machine learning techniques, including the Faster-RCNN model with the Inception-ResNet-v2 architecture for analyzing ultrasound breast images, have fine-tuned these procedures and demonstrate promise for integration into surgical practices. AI algorithms can enhance customized surgical approaches and provide predictions regarding patient-specific outcomes. When applied to reconstructive microsurgery, machine learning has the potential to predict flap perfusion and appropriate size based on perforator characteristics that may significantly reduce the amount of intraoperative decision-making needed as well as potentially improve outcomes such as partial flap necrosis due to incorrect perforator selection or oversized flaps.

Ultrasonography has a known learning curve and is not widely integrated into plastic surgery training [68]. This inherent user dependency of ultrasonography for image acquisition and interpretation presents a challenge to more consistent utilization of the technology, in addition to common barriers such as cost and equipment availability. A 2021 systematic review by Cho et al. noted that most of the literature on ultrasound in plastic surgery was for non-reconstructive procedures [8]. A critical consideration for the more widespread utilization of ultrasonography includes the ability for surgeons to easily learn and apply the technology with good reproducibility. Malagon et al. demonstrated an overall 60% correct responses rate in plastic surgeons identifying anatomic structures on previously obtained anatomic images; however, more complex but pertinent structures such as lymphatic vessels had lower scores [69]. In addition, the study only tested image identification and not image acquisition, the latter of which is likely the most challenging aspect of ultrasonography. While more studies are needed, the increasing literature on ultrasound in reconstructive microsurgery and plastic surgery points to a bright future for this technology within the specialty. 

## 4. Conclusions

The utilization of ultrasound technology in reconstructive microsurgery, particularly in free tissue transfer and lymphovenous bypass procedures, offers a multitude of benefits and underscores its significance in modern surgical practice. Color duplex ultrasonography allows for the precise visualization and assessment of perforator flow in real time, allowing for efficient design of perforator flaps. The ability to visualize small perforators and precisely map their course throughout the subcutaneous tissue also opens the door for more efficient planning and execution of thin, superthin and freestyle flaps. Similarly, the advent of clinically accessible UHFUS provides unparalleled visualization of lymphatic channels and venules that has revolutionized LVB surgery. Multiple additional uses of ultrasound intraoperatively and postoperatively have also been described. In the end, ultrasound is just another tool for the reconstructive microsurgeon. However, when appropriately utilized, the literature has demonstrated its ability to improve upon planning and efficiency and thereby our overall care of patients in reconstructive microsurgery. Overall, the integration of ultrasound technology in microsurgical procedures demonstrates its indispensable role in advancing surgical precision, improving patient outcomes, and shaping the future of reconstructive surgery.

## Data Availability

No new data were created or analyzed in this study. Data sharing is not applicable to this article. A.S. is a research consultant for Abbvie, Inc.
